# Development of artificially intelligent tool for analysis and prediction of myopia progression among school-going children

**DOI:** 10.1186/s13063-026-09648-w

**Published:** 2026-04-14

**Authors:** Priti Singh, Vidhya Verma, Samendra Karkhur, Arushi Beri, Jyoti Singhai, Dheraj Kumar Agrawal

**Affiliations:** 1 All India Institute of Medical Sciences, Bhopal, India; 2 Department of Electronics and Communication Engineering, Maulana Azad National Institute of Technology, Bhopal, India

**Keywords:** Myopia progression, School children, Artificial intelligence, Machine learning, Predictive modeling, Vision screening, Public health ophthalmology

## Abstract

**Background:**

Myopia is a growing public health concern among school-going children, with increased screen time and reduced outdoor activities contributing to its early onset and rapid progression. In India, the absence of a standardized, population-based dataset reflecting socio-economic and environmental diversity limits the development of accurate prediction tools. This study aims to design and validate an artificial intelligence (AI)-based model to analyze and predict myopia progression among children aged 6 to 18 years in urban and rural areas.

**Methods:**

This is a prospective, community-based study conducted in two phases across five districts of Bhopal division, India. Phase one involves a cross-sectional survey of approximately 5,000 school children to collect demographic and ocular health data, including visual acuity, refractive error, axial length, corneal parameters, and lifestyle factors. In phase two, a longitudinal cohort comprising 10% of the phase one participants (balanced for existing and non-existing myopia cases) will be followed every 6 months over 2 years. Machine learning algorithms, including linear regression, support vector machines, XGBoost, and deep learning models such as convolutional neural networks, will be trained on this dataset. An AI-based mobile application will be developed to enable field-level prediction and screening.

**Discussion:**

This study will generate the first large-scale Indian dataset on myopia progression among children, incorporating diverse socio-economic backgrounds. The validated AI model and mobile application will support early identification of at-risk children, optimize resource allocation, and inform national screening strategies. By integrating real-time data analytics with field-l.

**Trial registration:**

CTRI/2025/07/091243. Registered on 21/07/2025.

## Administrative information

**Table:** 

Title {1}	Development of Artificially Intelligent tool for analysis and prediction of myopia progression among school going children
Trial registration {2a and 2b}	CTRI/2025/07/091243
Protocol version {3}	Protocol Version: Version 1.0 Date: 16 July 2025
Funding {4}	This study is funded by the Indian Council of Medical Research (ICMR) under the Investigator-Initiated Research Proposals for Small Extramural Grants scheme (Proposal ID: IIRPSG-2024-01−03524). The financial support covers personnel, equipment, travel, data collection, and software development costs over a 36-month project period Material and technical support is provided by: • All India Institute of Medical Sciences (AIIMS), Bhopal – for clinical expertise, participant recruitment, and on-site examinations • Maulana Azad National Institute of Technology (MANIT), Bhopal – for AI model development, data analysis, and mobile application development through access to high-performance computing facilities No commercial sponsorship or third-party funding has been received
Author details {5a}	1 Dr. Priti Singh Additional Professor All India Institute of Medical Sciences, Bhopal priti.ophtho@aiimsbhopal.edu.in 9993896479 Permanent PI 2 Dr. Vidhya Verma Additional Professor All India Institute of Medical Sciences, Bhopal drvidhya301@gmail.com 9754461679 Permanent Co-PI 3 Dr. Samendra Karkhur Associate Professor All India Institute of Medical Sciences, Bhopal karkhurs@gmail.com 9914853225 Permanent Co-PI 4 Dr. Arushi Beri Project Research Scientist – Medical All India Institute of Medical Sciences, Bhopal arushi.beri25@gmail.com 5 Prof. Jyoti Singhai Professor Maulana Azad National Institute of Technology j.singhai@gmail.com 9425028222 Permanent Co-PI 6 Dr. Dheraj Kumar Agrawal Associate Professor Maulana Azad National Institute of Technology profdheerajagrawal@gmail.com 7869301930 Permanent Co-PI
Name and contact information for the trial sponsor {5b}	Indian Council of Medical Research (ICMR) Department of Health Research Ministry of Health and Family Welfare, Government of India V. Ramalingaswami Bhawan, Ansari Nagar, New Delhi – 110029, India Website: https://www.icmr.gov.in Sponsor Contact Person: Dr. Suruchi Aggarwal Scientist-C (Coordinator – Extramural Research Programme) Department of Health Research Indian Council of Medical Research Email: suruchia.hq@icmr.gov.in | Phone: + 91 9821900689
Role of sponsor {5c}	The Indian Council of Medical Research (ICMR), as the funding agency, provides financial support for the conduct of this study under the Investigator-Initiated Research Proposals for Small Extramural Grants scheme. The sponsor has no role in the study design; collection, management, analysis, or interpretation of data; writing of the report; or the decision to submit the report for publication. These responsibilities lie solely with the principal investigator and the research team. The investigators retain full autonomy over the conduct and dissemination of the research

## Introduction

### Background and rationale {6a}

Myopia, or near-sightedness, has emerged as a significant global public health issue, particularly among school-aged children. The rising prevalence of myopia has been linked to increased screen time, reduced outdoor exposure, and behavioral changes following the COVID-19 pandemic. In India, there is a pressing need to address this trend, especially in light of the country’s diverse socio-economic and environmental landscape [1].

Recent global studies have shown that early identification and intervention can slow myopia progression, reducing the risk of severe ocular complications such as retinal detachment, glaucoma, and macular degeneration later in life [2]. Traditional methods of screening and monitoring, however, are often resource-intensive and dependent on access to ophthalmic specialists, which limits large-scale deployment in low-resource settings [3–5].

Artificial intelligence (AI) offers a promising avenue to address these challenges. AI models have demonstrated high accuracy in classifying and predicting ocular diseases based on both imaging and non-imaging datasets. For instance, Foo et al. [6] highlighted the potential of AI in identifying refractive errors using fundus photography, while Li et al. [7] emphasized AI’s role in personalized risk assessment for myopia progression. Additionally, research by Zhang et al. [8] showed that AI algorithms can detect subtle changes in axial length and refractive parameters that may precede clinical diagnosis [9].

Despite these advances, there is a conspicuous lack of standardized Indian datasets to train and validate such models. Most existing models are trained on non-Indian populations and may not account for local variations in genetics, environment, or socio-economic factors. Furthermore, no comprehensive AI-based mobile application currently exists for school-based screening of myopia progression in Indian children.

This study is therefore designed to fill these critical gaps by:

- Creating a large-scale, representative dataset of ocular and demographic parameters from school-going children across urban and rural areas;
- Developing and validating AI-based predictive models using both conventional machine learning and deep learning techniques;
- Integrating the validated model into a mobile application to enable field-level deployment for early detection and intervention.

The initiative aligns with national health priorities by promoting preventive pediatric ophthalmology and leveraging digital health tools to reduce the burden on tertiary care facilities.

### Objectives {7}

#### Primary objective

- To develop and validate an artificial intelligence (AI)-based predictive model for assessing myopia progression among school-going children aged 6–18 years, based on demographic, environmental, and ocular health parameters.

#### Secondary objectives

1. To systematically collect and analyse cross-sectional and longitudinal data on myopia-related risk factors in children from diverse socio-economic backgrounds in urban and rural areas of the Bhopal division.
2. To identify and rank key predictors (e.g., axial length, screen time, parental myopia) contributing to myopia progression using machine learning algorithms.
3. To design and deploy a mobile application embedded with the AI model to facilitate field-level risk prediction and early screening without the need for specialist intervention.
4. To conduct awareness programs aimed at the prevention and early intervention for myopia progression in school children.

##### ***Hypothesis***

Among school-going children aged 6–18 years, AI-based analysis of ocular and behavioral parameters can accurately predict the risk of myopia progression and serve as a scalable tool for early detection and preventive ophthalmic care in diverse community settings.

### Trial design {8}

This study is designed as a prospective, community-based, observational cohort trial conducted in two sequential phases:

- Phase 1: A cross-sectional, single-group observational survey of approximately 5,000 school-going children (ages 6–18) across rural and urban regions of the Bhopal division. This phase aims to establish baseline demographic, environmental, and ocular health data.
- Phase 2: A longitudinal, single-group cohort follow-up involving approximately 500 children (10% of the initial sample), stratified into myopic and non-myopic groups. Participants will be re-evaluated every 6 months over a 24-month period to monitor changes and assess myopia progression.

There is no randomization or allocation to intervention groups, as this is a non-randomized, single-arm exploratory study. The study framework is exploratory, aimed at developing and validating an AI-based risk prediction model rather than comparing clinical interventions. This study is classified as minimal risk, non-interventional observational cohort study. The procedures involved—including visual acuity testing, auto-refraction, axial length measurement, fundus photography, and questionnaire-based data collection—are standard, non-invasive, and routinely performed in school eye screening programs.

## Methods: participants, interventions, and outcomes

### Study setting {9}

This study will be conducted in a community-based school setting across both rural and urban regions of the Bhopal division, located in the state of Madhya Pradesh, India. The Bhopal division comprises five districts: Bhopal, Raisen, Rajgarh, Sehore, and Vidisha.

Data will be collected from government and private schools representing diverse socio-economic backgrounds. Ocular examinations and survey-based assessments will be conducted on-site in schools by field teams comprising ophthalmologists, optometrists, and trained research personnel from All India Institute of Medical Sciences (AIIMS), Bhopal, with technical support from Maulana Azad National Institute of Technology (MANIT), Bhopal.

All study activities will be based in India, and no data collection is planned outside the country.

A complete list of participating schools and study sites can be obtained from the Principal Investigator upon reasonable request. This information is also available in the internal project implementation plan approved by the Institutional Ethics Committee of AIIMS Bhopal.

### Eligibility criteria {10}

#### Inclusion criteria (participants)

- School-going children aged 6 to 18 years
- Enrolled in selected government or private schools in urban or rural areas of Bhopal division (Bhopal, Raisen, Rajgarh, Sehore, Vidisha)
- Children whose parents or legal guardians provide written informed consent and who provide age-appropriate assent
- Willing to participate in periodic ocular evaluations and follow-up over a 2-year period (for longitudinal phase)
- Children are considered a vulnerable pediatric population; therefore, inclusion is permitted only with written informed parental/legal guardian consent and age-appropriate child assent in accordance with ICMR National Ethical Guidelines (2017). Additional safeguards, including simplified explanations and the right to withdraw at any time, will be ensured.

#### Exclusion criteria (participants)

- Children with a history of ocular trauma, congenital eye anomalies, or prior ocular surgery
- Children with known systemic conditions affecting ocular health (e.g., diabetes, neurological disorders)
- Children unwilling or unable to cooperate with ocular examinations or data collection procedures
- Children already enrolled in any other interventional ophthalmic study
- Children who express unwillingness, discomfort, or dissent to participate at any stage of the study, even if parental or guardian consent has been obtained

#### Eligibility criteria for study centers

- Schools located within the five districts of the Bhopal division
- Institutions willing to provide administrative permission and cooperate with data collection activities
- Schools representing diverse socio-economic backgrounds, ensuring representation of urban, rural, and tribal populations

#### Eligibility criteria for study personnel

- Ophthalmologists, optometrists, and trained research assistants from AIIMS Bhopal are responsible for clinical evaluation and data collection
- AI and data science experts from MANIT Bhopal involved in model development, application programming, and data analysis
- All personnel involved in data collection and analysis must have completed ethics and research conduct training and be authorized under the Institutional Ethics Committee protocol
- As this study involves minors (6–18 years), who constitute a vulnerable population under national and international ethical frameworks, special protections will be implemented Participation will be entirely voluntary. If a child refuses or withdraws assent at any point, their decision will be respected without penalty, irrespective of prior parental consent. No coercion, inducement, or academic influence will be used to encourage participation.

### Who will take informed consent? {26a}

Informed consent will be obtained from the parents or legal guardians of all participating children prior to enrolment in the study. In addition, age-appropriate assent will be obtained from children aged 7 years and above, in accordance with ethical guidelines for paediatric research.

- Provision of the Participant Information Sheet at least 48–72 h prior to enrolment
- Conduct of consent procedures by trained research personnel independent of school authorities and not involved in academic evaluation

The process will be coordinated and overseen by trained research staff and ophthalmologists from AIIMS Bhopal. Consent forms will be distributed in the local language (Hindi) as well as in English, and detailed verbal explanations will be provided during parent–teacher meetings or scheduled school visits. The study team will ensure that guardians fully understand the study objectives, procedures, potential risks, and data privacy measures before signing.

For children selected for the longitudinal follow-up cohort, consent will be re-confirmed at each follow-up visit.

All consent and assent documents have been reviewed and approved by the Institutional Human Ethics Committee (IHEC) of AIIMS Bhopal, and signed forms will be securely stored in compliance with data protection regulations.

In addition to written informed consent from parents or legal guardians, age-appropriate assent will be obtained from participating children in accordance with the ICMR National Ethical Guidelines for Biomedical and Health Research Involving Human Participants (2017).

Assent procedures will be tailored to developmental capacity:

- Children aged 7–11 years**:** A simplified verbal explanation using child-friendly language will be provided, supported by a brief illustrated information sheet in Hindi and English. The study purpose, procedures (vision testing, eye drops, measurements), and the voluntary nature of participation will be explained. Verbal assent will be documented by the investigator.
- Children aged 12–18 years**:** A written assent form, separate from the parental consent form, will be provided. The form will describe the study objectives, procedures, possible discomfort (e.g., temporary blurring after cycloplegic drops), confidentiality safeguards, and the right to withdraw at any time without academic or medical consequences. Written assent will be obtained and retained in study records.

Children will be given adequate time to ask questions. The explanation will be delivered in a non-coercive environment, without the presence of school authorities influencing the decision.

If a child declines participation or withdraws assent at any stage, their decision will be respected unconditionally, even if parental consent has been granted. Such children will not face any academic disadvantage, and no further study-related procedures will be performed.

To prevent therapeutic misconception, it will be explicitly explained that this is an observational research study and not a therapeutic intervention. Participation does not guarantee medical benefit beyond routine eye screening, and refusal will not affect access to standard clinical care, school services, or academic standing.

### Additional consent provisions for collection and use of participant data and biological specimens {26b}

This study does not involve the collection of biological specimens. However, coded ocular health and demographic data collected during the main study may be used in future ancillary analyses, including algorithm refinement, external validation studies, and related non-commercial academic research projects.

Participants and their legal guardians will be informed of this possibility during the consent process. A specific clause is included in the informed consent form requesting permission for:

- Future use of de-identified data for related studies approved by the ethics committee
- Data sharing with collaborating academic institutions under appropriate data sharing agreements
- Storage of coded data in a secure, password-protected institutional database for a maximum duration of 10 years

## Interventions

This is a non-interventional, observational cohort study; therefore, no clinical or therapeutic intervention will be administered to participants as part of the trial.

The “intervention” in this study pertains to the development and application of an artificial intelligence (AI)-based predictive model for myopia progression, which will utilize data collected through non-invasive, standard ophthalmic assessments and structured questionnaires. All procedures are observational and diagnostic in nature and include:

- Visual acuity testing (unaided and best-corrected)
- Cycloplegic auto-refraction
- Axial length and corneal thickness measurements
- Fundus photography
- Lifestyle and demographic data collection (screen time, outdoor activity, dietary habits, parental myopia history, etc.)

These procedures will be conducted by trained ophthalmologists and optometrists using validated, portable instruments. No drugs, therapeutic regimens, or experimental clinical procedures will be introduced as part of this study.

The AI model and associated mobile application will be applied to collected data retrospectively and prospectively for prediction purposes only, without influencing or altering standard clinical care pathways for participants.

### Explanation for the choice of comparators {6b}

This is an observational cohort study designed to develop and validate an artificial intelligence (AI)-based prediction model for myopia progression. As such, the study does not involve therapeutic or diagnostic interventions requiring active comparators.

However, within the context of AI model development and validation, internal comparisons will be made between:

- Children with pre-existing myopia and those with no baseline myopia (to identify predictive differences)
- Multiple machine learning algorithms (e.g., linear regression, support vector machines, XGBoost, deep learning) to determine the most accurate predictive model
- AI-based predictions versus clinical assessments and historical progression data during longitudinal follow-up to evaluate predictive accuracy and sensitivity

These internal comparators are essential for optimizing model performance but do not involve assigning participants to treatment or intervention arms. The study is exploratory in nature and does not test the superiority or inferiority of any clinical intervention.

### Intervention description {11a}

This is an observational study. No clinical interventions are administered. All assessments are non-invasive diagnostics.

### Criteria for discontinuing or modifying allocated interventions {11b}

This is an observational study. No clinical interventions are administered. All assessments are non-invasive diagnostics.

### Strategies to improve adherence to interventions {11c}

This is an observational study. No clinical interventions are administered. All assessments are non-invasive diagnostics.

### Relevant concomitant care permitted or prohibited during the trial {11d}

This is an observational study. No clinical interventions are administered. All assessments are non-invasive diagnostics.

### Provisions for post-trial care {30}

This study involves non-invasive observational assessments and does not include administration of any therapeutic or experimental clinical interventions. As such, the risk of physical or psychological harm to participants is minimal.

However, in accordance with national ethical guidelines and institutional policy:

- Any child identified during the study as having previously undiagnosed or untreated refractive errors or ocular conditions will be referred to the Department of Ophthalmology at AIIMS Bhopal or a nearby government ophthalmic center for appropriate clinical evaluation and care, free of cost under available public health schemes.
- No post-trial intervention or follow-up treatment is mandated beyond the scheduled study assessments, as no interventional product or device is being tested.
- In the unlikely event of harm resulting directly from participation in study procedures (e.g., adverse reaction to cycloplegic drops), immediate medical attention will be provided at AIIMS Bhopal, and the participant will be eligible for care and compensation in accordance with the Ethical Guidelines for Biomedical Research on Human Participants (ICMR, 2017) and the guidelines of the Institutional Ethics Committee (IEC).

### Outcomes {12}

#### Primary outcome

- Measurement Variable: Risk of myopia progression
- Analysis Metric: Change in spherical equivalent refraction (SER) and axial length over time
- Method of Aggregation: Mean change from baseline to each 6-month follow-up, stratified by age group, urban/rural location, and baseline refractive status
- Time Points: Baseline, 6 months, 12 months, 18 months, and 24 months
- Clinical Relevance**:** Monitoring SER and axial elongation is clinically validated as the gold standard for quantifying myopia progression. Accurate prediction supports early intervention and reduces the risk of long-term complications such as retinal detachment and macular degeneration
- The AI model performance metrics (e.g., AUROC, sensitivity, specificity) will be evaluated against the primary clinical outcome of myopia progression (change in spherical equivalent refraction and axial length), thereby linking predictive accuracy directly to clinically meaningful disease progression

#### Secondary outcomes

1. AI Model Performance Metrics
  o Measurement Variables: Accuracy, sensitivity, specificity, F1 score, AUROC (Area Under Receiver Operating Characteristic Curve)
  o Analysis Metric: Model performance against test data
  o Method of Aggregation: Aggregated over cross-validation folds and independent test sets
  o Time Point: After complete data collection at each longitudinal stage
  o Clinical Relevance: Demonstrates the utility of AI for triage and risk stratification without immediate clinical intervention
2. Identification of Dominant Risk Factors
  o Measurement Variables: Screen time, parental myopia, near work duration, outdoor activity, corneal curvature, axial length, etc.
  o Analysis Metric: Feature importance scores and multivariate regression coefficients
  o Method of Aggregation: Ranked predictive power in final AI model
  o Time Point: End of Phase 1 (cross-sectional analysis)
  o Clinical Relevance: Supports public health planning and targeted interventions
3. Mobile Application Usability and Accuracy
  o Measurement Variables: Accuracy of app-based predictions compared to clinical diagnoses
  o Analysis Metric: Positive predictive value, user-reported ease-of-use scores
  o Method of Aggregation: Mean values and usability ratings (Likert scale)
  o Time Point: Pilot deployment at 18 months
  o Clinical Relevance: Determines the viability of a scalable school-level screening tool

#### Exploratory outcomes

- Awareness and behavior change: Number of students reached and qualitative feedback on myopia prevention education initiatives
- Data Quality Indicators: Completeness and reliability of longitudinal data across follow-up visits

### Participant timeline {13}

Participants will be enrolled and followed up over a 36-month period as per the schedule below:

#### Phase 1: baseline cross-sectional survey (months 1–12)

- Enrolment and Consent: Months 1–3
- Baseline Assessment (Ocular and Demographic): Months 2–9
- Initial Data Cleaning and Preprocessing: Months 6–12
- AI Model (v1) Training on Baseline Data: Months 9–12

#### Phase 2: longitudinal follow-up cohort (months 13–36)

- Cohort Selection (10% of Phase 1): Month 13
- Follow-up Assessments:
  o Visit 1: Month 18
  o Visit 2: Month 24
  o Visit 3: Month 30
  o Visit 4: Month 36
- AI Model Refinement: Ongoing after each follow-up round
- Mobile App Pilot Testing: Month 30–36

**Schematic diagram of study timeline**

**Table:** 

Study period	Enrolment	Baseline (T0)	Follow-up 1 (T1)	T2	T3	T4
Time point (months)	0–3	2–9	18	24	30	36
Informed consent	✔					
Eligibility assessment	✔					
Demographic data		✔				
Ocular examinations		✔	✔	✔	✔	✔
Questionnaire		✔	✔	✔	✔	✔
AI model training		✔	✔	✔	✔	✔
Mobile App testing					✔	✔

### Sample size {14}

The estimated number of participants required to achieve the study’s objectives is 5,000 school-going children for the baseline cross-sectional phase, with a 10% subcohort (approximately 500 children) followed longitudinally over a period of 24 months.

#### Phase 1: cross-sectional component

A minimum sample size of 1640 children was initially calculated based on:

- Expected incidence rate of myopia: 3.4% (as per Saxena et al., 2017; PLoS ONE, North India Myopia Study)
- Confidence level: 95% (*Z* = 1.96)
- Precision (d): 2.5%

Sample size formula used:

N = Z2 × p × (1 − p)d2N = \frac{Z^2 \times p \times (1—p)}{d^2}N = d2Z2 × p × (1 − p)​ N = (1.96)2 × 0.034 × (1 − 0.034)(0.025)2≈1,484N = \frac{(1.96)^2 \times 0.034 \times (1—0.034)}{(0.025)^2} ≈ 1,484N = (0.025)2(1.96)2 × 0.034 × (1 − 0.034)​≈1,484.

Adjusting for a 10% non-response or attrition rate:

N≈1,640N ≈ 1,640N≈1,640

To increase statistical power and allow for subgroup analysis (by age, gender, urban/rural, socioeconomic status), the sample was scaled up to 5000 participants, ensuring diversity and enabling robust AI model training with stratified data.

#### Phase 2: longitudinal cohort

A subset of 10% (approx. 500 children) will be selected from Phase 1 using AI-based parameter selection and stratified sampling (equal representation of myopic and non-myopic participants). This sample size is adequate for:

- Detecting meaningful differences in myopia progression rates (in diopters and axial length)
- Training and validating AI models using longitudinal data with multiple follow-ups
- Ensuring sufficient statistical power (> 80%) for machine learning model performance metrics such as AUC, sensitivity, and specificity

Sample size assumptions were determined in consultation with biostatisticians and are justified based on the objectives of developing and validating a predictive AI tool, rather than testing clinical efficacy of an intervention.

#### Recruitment {15}

To ensure achievement of the target sample size of 5000 school-going children across urban and rural areas of the Bhopal division, the following strategies will be employed:

1. Institutional Collaboration:
  Formal letters of collaboration and administrative approvals have been obtained from district education authorities and individual schools to facilitate access to eligible students across five districts (Bhopal, Raisen, Rajgarh, Sehore, and Vidisha).
2. School-based outreach:
  Recruitment will be conducted through government and private schools identified via stratified sampling. Each school will be visited by the study team to conduct parent–teacher meetings, distribute participant information sheets, and explain the study process in local languages.
3. Awareness and engagement sessions:
  Educational sessions and eye health awareness activities will be conducted in schools to enhance participation. These sessions will inform parents and students about the importance of early myopia detection and the non-invasive nature of the assessments.
4. Dedicated field teams:
  Multiple trained field teams (ophthalmologists, optometrists, and research assistants) will be deployed simultaneously across districts to maximize enrolment efficiency and geographic coverage.
5. Flexible scheduling:
  Data collection visits will be coordinated with school administrators to minimize disruption to academic schedules and maximize attendance during assessment days.
6. Follow-up cohort retention:
  For the longitudinal phase, participants selected for follow-up will be enrolled with parental consent for periodic re-assessments every six months, with SMS/phone reminders and on-site visits planned to maintain cohort continuity.

These recruitment strategies are designed to ensure representative sampling, minimize attrition, and achieve timely enrolment within the project timeline.

## Assignment of interventions: allocation

### Sequence generation {16a}

This study does not involve random allocation to intervention or control groups, as it is a non-interventional observational cohort trial.

However, for the purpose of selecting a representative 10% sub-cohort (approximately 500 children) from the cross-sectional sample for longitudinal follow-up, a structured sampling process will be used:

- The allocation sequence for follow-up selection will be generated using a computerized stratified random sampling algorithm.
- Stratification factors will include:
  o Baseline myopia status (myopic vs. non-myopic)
  o Age group (6–10, 11–14, 15–18 years)
  o Geographic location (urban vs. rural)
  o Gender

This approach will ensure balanced representation across key demographic and clinical subgroups, thereby enhancing the generalizability of AI model training and validation.

The specific algorithm and code for allocation will be maintained by the data analysis team at MANIT Bhopal and will not be accessible to field teams or investigators responsible for participant enrolment to avoid selection bias.

### Concealment mechanism {16b}

As this is a non-randomized, observational cohort study, there is no allocation to therapeutic or intervention groups. Therefore, conventional allocation concealment (e.g., sealed envelopes or centralized randomization) is not applicable.

For the selection of the 10% longitudinal follow-up cohort, a stratified random sampling algorithm will be used, based on pre-specified criteria (myopia status, age group, gender, and geographic area). This sampling will be conducted centrally by the data analysis team at Maulana Azad National Institute of Technology (MANIT), Bhopal, using a computer-generated randomization process.

To minimize selection bias:

- The allocation sequence will be stored securely and will **not be accessible** to the field teams involved in school visits or data collection.
- The list of selected participants will be released **only after completion of baseline data collection**, ensuring that the initial assessments are conducted without any influence from the selection status.

This mechanism maintains methodological rigor and reduces the risk of conscious or unconscious selection bias in follow-up participant identification.

### Implementation {16c}

As this is an observational study without intervention groups, participants are not assigned to interventions. However, a selection process is used to identify a 10% sub-cohort from the baseline sample for longitudinal follow-up. The roles related to this process are as follows:

- Generation of the Allocation Sequence:
- The stratified random sampling algorithm used to select participants for follow-up will be developed and executed by the data science team at Maulana Azad National Institute of Technology (MANIT), Bhopal). The algorithm will be based on predefined strata (e.g., myopia status, age group, gender, and geographic region).
- Enrollment of Participants:
- Participants will be enrolled at the school level by trained ophthalmologists and research staff from AIIMS Bhopal, following informed consent from parents or legal guardians and assent from children (as applicable).
- Assignment to Longitudinal Follow-Up:
- The list of selected participants for the follow-up phase will be generated only after completion of baseline assessments, and those participants will be contacted by the field team from AIIMS Bhopal for scheduling periodic reassessments. The enrolment team will have no influence over the selection process.

This separation of roles ensures objectivity, prevents selection bias, and preserves the integrity of cohort formation.

### Assignment of interventions: blinding

#### Who will be blinded {17a}

This is an observational cohort study with no therapeutic or clinical interventions assigned; therefore, blinding of participants or care providers is not applicable.

However, to minimize bias and ensure objective analysis:

- Outcome Assessors (AI Developers/Data Scientists):
- The data analysis team at Maulana Azad National Institute of Technology (MANIT), Bhopal, responsible for developing and evaluating the AI prediction models, will be blinded to the identity of individual participants and their school affiliations. Only anonymized and coded datasets will be used for model training and validation.
- Field Investigators and Clinical Examiners:
- Field personnel conducting ocular examinations and data collection at schools will be unaware of which participants will be included in the longitudinal follow-up phase at the time of initial data collection. This is to prevent any differential treatment or attention.
- Participants:
- As no clinical intervention is administered and participants are not exposed to comparative groups, blinding of participants is not relevant.

This level of blinding is appropriate to the study design and ensures that both data collection and analysis are conducted with minimal risk of observational or analytical bias.

### Procedure for unblinding if needed {17b}

As this is a non-interventional, observational cohort study, no participants or care providers are blinded to a treatment assignment. Consequently, there is no formal “allocation” to be unblinded and the standard unblinding procedures for clinical trials do not apply.

However, in the context of our data-analysis blinding—whereby the AI development team at MANIT works exclusively with anonymized, coded data—the following applies:

- Circumstances permitting re-identification (unblinding):
  1. Safety concerns: If an unexpected ocular finding during data analysis suggests an urgent need for clinical follow-up (e.g., detection of a sight-threatening condition), the coding key may be accessed by the Principal Investigator to identify and refer the participant for immediate care.
  2. Data verification or audit: For regulatory or ethical audits, the Sponsor or Ethics Committee may request linkage between coded data and participant identity to confirm compliance or investigate reported issues.
- Procedure for revealing participant identity:
  1. Authorization: Any request to access the coding key must be approved in writing by the Principal Investigator (Dr. Priti Singh) and the Institutional Ethics Committee of AIIMS Bhopal.
  2. Controlled Access: A designated data-management officer will retrieve the minimal necessary identifiers and provide them only to the clinical team for the stated purpose.
  3. Documentation: All unblinding events will be logged in the trial master file, including date, reason, personnel involved, and actions taken.

## Data collection and management

### Plans for assessment and collection of outcomes {18a}

#### Baseline and follow-up data collection

Data will be collected in two phases: a cross-sectional baseline assessment (Phase 1) and a longitudinal follow-up (Phase 2). Data collection will be performed on-site in schools across the Bhopal division (Madhya Pradesh, India) by trained field teams from AIIMS Bhopal.

1. Demographic and lifestyle data (questionnaire-based):
  Instruments: Structured, pre-tested questionnaires administered to parents/guardians and students (as age-appropriate)
  Variables collected: Age, gender, dietary habits, screen time, near work duration, outdoor activity, family history of myopia, socio-economic status
  Reliability/Validity: Based on instruments used in previously validated school eye health studies (e.g., AIIMS Delhi NIM Study); pre-tested in a local pilot survey.
2. Ocular assessments (clinical examination):
  Performed using portable, validated ophthalmic instruments by optometrists and ophthalmologists:
  Uncorrected Visual Acuity (UCVA) and Best Corrected Visual Acuity (BCVA): Standard Snellen chart
  Cycloplegic Auto-refraction: Handheld autorefractometer with keratometry (validated device)
  Axial Length and Corneal Thickness: Portable A-scan with pachymeter
  Corneal Curvature and Refraction Power: Recorded during auto-refraction
  Fundus Photography: Non-mydriatic portable fundus camera

All measurements will be conducted in duplicate where applicable (e.g., axial length) to ensure reliability, and any inconsistencies will be re-checked by a senior clinician on-site.

#### Follow-up assessments (every 6 months for 24 months)

Participants selected for longitudinal follow-up will undergo the same clinical and questionnaire-based assessments at each 6-month interval. Changes in spherical equivalent refraction and axial length will be primary outcome measures.

### Data quality assurance measures

- Training and standardization: All examiners will undergo centralized training by AIIMS Bhopal prior to field deployment; SOPs will be strictly followed
- Inter-observer calibration: Random sample reviews will be performed to assess inter-observer variability and maintain consistency
- Real-time data entry: Digital data entry using custom forms in a secure, encrypted platform with built-in range and consistency checks
- Daily field verification: On-site supervisors will verify 5% of randomly selected entries each day
- Data backup and auditing**:** Data will be backed up daily and subject to weekly audits by the data coordination team at MANIT

#### Data collection forms

Detailed Case Report Forms (CRFs) and questionnaires are available as supplementary materials to this protocol and have been submitted to the Institutional Ethics Committee. Electronic CRFs are programmed into the data capture platform to minimize manual errors.

### Plans to promote participant retention and complete follow-up {18b}

To minimize attrition and ensure high follow-up rates during the 24-month longitudinal phase, the following measures will be implemented:

1. School and parent engagement:
  o Ongoing collaboration with school principals and parent–teacher associations to maintain institutional support.
  o Educational sessions and feedback to parents regarding their child’s eye health to foster trust and continued participation.
2. Communication and reminders:
  o SMS and phone call reminders to parents prior to each follow-up visit.
  o Distribution of appointment cards at each visit, with the next scheduled date printed clearly.
3. Flexible scheduling and on-site visits:
  o Follow-up assessments will be planned during school hours at convenient times to avoid academic disruption.
  o Re-visits scheduled for absentees to ensure maximum data recovery.
4. Minimal invasiveness and incentives:
  o All assessments are non-invasive, encouraging cooperation.
  o Age-appropriate educational materials or certificates of participation will be provided as positive reinforcement.

#### Data collection for participants lost to follow-up

For participants who discontinue participation in the longitudinal follow-up:

- Last Available Ocular Data (e.g., visual acuity, axial length, spherical power) and baseline demographics will be retained for analysis.
- Reasons for discontinuation (e.g., transfer to another school, withdrawal of consent) will be documented.
- Wherever feasible, a final assessment will be attempted before exit, with parental consent.

Missing data will be addressed during statistical analysis using appropriate imputation techniques where required, as described in the statistical analysis plan.

### Data management {19}

#### Data entry and coding

- All data will be entered digitally at the point of collection using encrypted electronic case report forms (eCRFs) developed in collaboration with the data science team at MANIT Bhopal.
- Each participant will be assigned a unique anonymized identification** code** to maintain confidentiality.
- **Range and logic checks** will be built into the data entry interface to flag missing, out-of-range, or inconsistent responses in real-time.
- For select critical variables (e.g., axial length, visual acuity), duplicate entry and verification will be performed to minimize transcription errors.

#### Data security and storage

- Data will be stored on a secure, password-protected institutional server with access restricted to authorized personnel only.
- Regular automated daily backups will be performed to ensure data redundancy.
- Access to the identifiable dataset (e.g., name, school, contact) will be strictly limited to the Principal Investigator and designated data managers for follow-up coordination.
- All field devices (e.g., tablets or laptops) used for data collection will be encrypted and configured to auto-wipe in case of a security breach.

#### Quality assurance procedures

- A data monitoring committee comprising members from AIIMS Bhopal and MANIT will conduct monthly audits of 5–10% of data entries.
- A data dictionary and standard operating procedures (SOPs) for data handling have been developed and shared with all data collection teams.
- Discrepancies or missing values identified during audit will be queried and resolved within 5 business days.

#### Location of detailed procedures

The complete Data Management Plan (DMP), including SOPs for data handling, coding structure, and version control protocols, is maintained as an internal document and is available upon request from the Principal Investigator.

### Confidentiality {27}

All personal information of participants—including names, contact details, school identifiers, and medical data—will be collected, stored, and managed in strict accordance with ethical standards and institutional data protection protocols to ensure confidentiality before, during, and after the study.

#### Collection and handling

- Identifiable data (e.g., name, school, phone number) will be collected only once at enrolment, solely for the purpose of informed consent, participant tracking, and follow-up coordination.
- Each participant will be assigned a unique study ID code, which will be used throughout the data collection and analysis process to ensure pseudonymization.
- All physical documents (e.g., signed consent forms) will be stored in locked cabinets at AIIMS Bhopal, accessible only to authorized study personnel.

#### Storage and protection

- Electronic data will be stored on secure, password-protected servers managed by the research IT team at MANIT Bhopal.
- Identifiable information will be stored separately from de-identified clinical and questionnaire data.
- Access to identifiable data will be restricted to the Principal Investigator, data managers, and follow-up coordination team.

#### Sharing and dissemination

- Only de-identified, aggregate-level data will be used for publication, presentation, or model training.
- Any data shared with collaborators or third-party academic institutions will be governed by data sharing agreements and will exclude personally identifiable information.

#### Post-trial confidentiality

- All identifiable data will be retained for a period of 10 years post-trial in secure archives, after which it will be disposed of in accordance with institutional and national data retention policies.
- Participants will be informed of these procedures during the consent process.

### Summary of data flow

Data will be collected at school sites using electronic case report forms, encrypted and transmitted to a secure central server. Following cleaning and validation, de-identified datasets will be processed by the MANIT data science team for feature engineering, model training, and cross-validation. The best-performing model will then be integrated into a mobile application for field-level deployment, where predictions will be generated using real-time input data without altering standard clinical care.

These measures are designed to uphold the principles of confidentiality and data integrity as outlined in the ICMR Ethical Guidelines (2017) and GDPR-aligned best practices for data protection in research.

### Plans for collection, laboratory evaluation and storage of biological specimens for genetic or molecular analysis in this trial/future use {33}

This study does not involve the collection, storage, or laboratory evaluation of biological specimens for genetic or molecular analysis. All data collected will be observational, comprising non-invasive ocular measurements, demographic information, and behavioral risk factors related to myopia progression.

Accordingly:

- No biological samples (e.g., blood, saliva, or tissue) will be obtained from participants.
- No laboratory-based genetic or molecular testing is planned as part of the current study protocol or any ancillary study.
- All predictive analyses will rely solely on imaging (e.g., fundus photography) and structured clinical and environmental datasets.

## Statistical methods

### Statistical methods for primary and secondary outcomes {20a}

#### Primary outcome analysis

- Objective: To quantify and predict myopia progression over time based on spherical equivalent refraction (SER) and axial length measurements.
- Approach:
  o Descriptive statistics (mean, standard deviation) will be used to report baseline characteristics.
  o Linear mixed-effects models will be applied to assess longitudinal changes in SER and axial length over follow-up periods, accounting for repeated measures and potential confounders (e.g., age, screen time, parental myopia).
  o Time-to-event analysis (e.g., Kaplan–Meier estimates) may be used to assess time to clinically significant myopic progression (e.g., ≥ –0.50 D/year), if applicable.

#### Secondary outcome analysis

1. AI Model Performance Metrics:
  o Algorithms including linear regression, support vector machines, XGBoost, random forests, and deep learning models (e.g., CNNs) will be trained and validated.
  o Evaluation metrics: Accuracy, sensitivity, specificity, F1 score, AUROC, AUPRC (as appropriate for classification tasks).
  o Cross-validation techniques (e.g., 5-fold or 10-fold) will be used to ensure robustness and prevent overfitting.
2. Identification of Dominant Risk Factors:
  o Multivariable logistic regression and feature importance analysis (e.g., SHAP values for tree-based models) will identify significant predictors of myopia progression.
  o Interaction effects (e.g., between screen time and outdoor activity) will be explored.
3. Mobile Application Validation:
  o Agreement between app-predicted and clinician-confirmed cases will be assessed using Cohen’s kappa coefficient.
  o Usability scores from field users will be analyzed using descriptive statistics and Likert scale summaries.

#### Handling of missing data

- Multiple imputation or model-based methods will be used to address missing values, assuming data is missing at random.
- Sensitivity analyses will be performed to assess the impact of missingness on model accuracy and prediction.

#### Software:

- Statistical analyses will be performed using R, Python (scikit-learn, TensorFlow), and SPSS, as applicable.

#### Reference to statistical analysis plan (SAP)

A detailed statistical analysis plan (SAP), including model selection criteria, tuning parameters, and subgroup analysis strategies, is maintained as a separate document by the data science team at MANIT Bhopal and is available upon request.

### Interim analyses {21b}

No interim analyses are planned as the study is non-interventional. However, AI model development will occur iteratively after each follow-up phase to refine predictive accuracy.

### Methods for additional analyses (e.g., subgroup analyses) {20b}

The study includes planned subgroup analyses and adjusted analyses to enhance model performance interpretation and to explore differential effects across population segments.

#### Subgroup analyses

The following predefined subgroups will be analyzed to assess variability in myopia progression and predictive model performance:

- Age groups: 6–10 years, 11–14 years, 15–18 years
- Geographic location: Urban vs. rural participants
- Gender: Male vs. female
- Baseline myopia status: Myopic vs. non-myopic at enrolment.
- Parental myopia: Present vs. absent

Subgroup analyses will involve stratified modeling and comparative evaluation of outcome trends using interaction terms within regression models.

#### Adjusted analyses

Multivariable regression models (e.g., linear or logistic regression for continuous or categorical outcomes, respectively) will be adjusted for potential confounders including:

- Age
- Gender
- Screen time
- Duration of outdoor activity
- Socio-economic status
- Parental myopia

These adjustments aim to isolate the effect of independent predictors on myopia progression and improve the interpretability and generalizability of AI-based predictions.

#### Model sensitivity and robustness checks

- Internal cross-validation (e.g., k-fold) and bootstrapping will be performed.
- Sensitivity analysis will test the impact of varying thresholds for clinically significant myopia progression (e.g., ≥ –0.50 D/year vs. ≥ –1.00 D/year).
- Sub-models excluding outliers or high-leverage data points will be analyzed to assess stability.

These additional analyses will support the rigor, reproducibility, and real-world applicability of the developed AI model.

### Methods in analysis to handle protocol non-adherence and any statistical methods to handle missing data {20c}

#### Analysis population

All participants enrolled and assessed at baseline will be included in the intention-to-observe population, which includes:

- All participants with complete baseline demographic and ocular data
- All participants selected for longitudinal follow-up, regardless of the number of follow-up assessments completed

There is no randomization or treatment allocation; hence, conventional “per-protocol” or “as-randomized” classifications do not apply. However, for robustness, analyses will distinguish between:

- Full analysis set (FAS): All eligible participants with at least baseline data
- Complete-case analysis set (CCAS): Subset of participants with complete data at all scheduled time points
- Longitudinal analysis set: All participants in the follow-up cohort with at least one post-baseline visit

#### Handling of missing data

To address potential loss to follow-up and incomplete entries:

- Descriptive statistics will be used to assess the extent and pattern of missingness (MCAR, MAR, or MNAR)
- If data are assumed missing at random (MAR):
  o Multiple imputation will be used for demographic, behavioral, and ocular parameters
  o Imputation models will incorporate all relevant covariates to preserve statistical power and reduce bias
- If data are not missing at random (MNAR):
  o Sensitivity analyses will be conducted using worst-case and best-case scenarios
  o Missingness indicators may be included as covariates in predictive models

All imputed datasets will be pooled using Rubin’s rules, and imputation diagnostics will be documented as part of the statistical analysis plan.

### Plans to give access to the full protocol, participant level-data and statistical code {31c}

The research team is committed to transparency and knowledge dissemination in accordance with national and international research ethics standards.

- Full Protocol: A de-identified version of the final approved study protocol will be made publicly available via institutional repositories or published as a supplementary file alongside the primary results manuscript.
- Participant-Level Dataset: De-identified, aggregated participant-level data may be made available for academic research purposes upon reasonable request to the Principal Investigator, subject to approval by the Institutional Ethics Committee (IEC) at AIIMS Bhopal and execution of a formal data-sharing agreement. No identifiable personal data will be shared under any circumstances.
- Statistical Code and Algorithms: Statistical scripts and model code (e.g., for machine learning and AI-based prediction algorithms) developed during this study will be shared through open-access repositories such as GitHub or Zenodo following publication of primary results, where feasible and in compliance with institutional policies.

## Oversight and monitoring

### Composition of the coordinating centre and trial steering committee {5d}

The TSC will meet quarterly throughout the duration of the study (36 months), with additional meetings as needed.

The data team will coordinate with the AIIMS team on a monthly basis and as required during key model deployment phases.

1. Coordinating center
  The coordinating center for the trial is based at the Department of Ophthalmology, All India Institute of Medical Sciences (AIIMS), Bhopal. This center is responsible for the day-to-day management and operational execution of the study. Core responsibilities include:
  Participant enrolment coordination and school liaison
  Training and deployment of field data collection teams
  Oversight of informed consent, data collection, and clinical evaluations
  Logistics planning for school visits and follow-ups
    The coordinating centre will meet bi-weekly during the first 6 months of enrolment and monthly thereafter, with emergency meetings convened as required.
2. Trial Steering Committee (TSC)
  The Trial Steering Committee will provide overall scientific oversight and strategic direction. It will consist of senior clinical investigators, biostatisticians, and external advisors. Key responsibilities include:
  Reviewing trial progress and protocol adherence
  Advising on protocol amendments if necessary
  Monitoring recruitment, data completeness, and safety concerns
  Approving major publications or data sharing
3. Data Management and AI Modelling Team
  This team, based at Maulana Azad National Institute of Technology (MANIT), Bhopal, is responsible for:
  Development and maintenance of the data capture platform
  Data cleaning, validation, and database locking procedures
  AI model training, testing, and performance evaluation
  Maintaining data security and compliance with SOPs
4. Endpoint adjudication and clinical review committee
  A panel of ophthalmologists from AIIMS Bhopal will serve as the clinical adjudication team to:
  Verify clinical classifications of myopia and progression status
  Review and confirm key ocular measurements flagged by automated systems
  Resolve discrepancies or borderline cases
    The adjudication committee will convene biannually or upon review of a substantial batch of flagged data.
5. Ethics and regulatory oversight
  The Institutional Ethics Committee (IEC) at AIIMS Bhopal provides independent oversight. All major amendments, safety concerns, and reports will be submitted to the IEC in compliance with regulatory requirements.

### Composition of the data monitoring committee, its role and reporting structure {21a}

Given the non-interventional and minimal-risk nature of this observational cohort study, which does not involve clinical treatments or investigational drugs/devices, a formal independent Data Monitoring Committee (DMC) has not been constituted.

#### Justification for not establishing a DMC

- The study involves non-invasive, routine ophthalmic assessments and questionnaire-based data collection.
- No investigational product, therapeutic intervention, or random allocation is involved.
- The trial poses no foreseeable risks beyond standard visual screening procedures.
- Adverse event monitoring in the traditional sense is not applicable.

#### Internal oversight provisions

To maintain data quality and ethical integrity, internal oversight will be ensured through:

- Monthly internal data reviews by the data coordination team at MANIT Bhopal and AIIMS Bhopal
- Quarterly reporting to the Trial Steering Committee (TSC), which includes independent academic and clinical advisors
- Regular updates and compliance reporting to the Institutional Ethics Committee (IEC) at AIIMS Bhopal

These mechanisms are deemed adequate for ensuring participant safety, data reliability, and protocol adherence in this observational, low-risk study context.

### Adverse event reporting and harms {22}

An independent Clinical Safety Oversight Subcommittee under the Trial Steering Committee (TSC), comprising senior clinicians not involved in routine data collection, will review all adverse events. Serious adverse events (SAEs) will be reviewed within 48 h and reported to the Institutional Ethics Committee (IEC) as per regulatory timelines. The committee will oversee causality assessment, ensure timely medical or surgical management at AIIMS Bhopal if required, and document all actions in the Trial Master File.

#### Nature of study and risk profile

This study is strictly observational and involves non-invasive ophthalmic assessments, questionnaires, and biometric measurements. No investigational products, therapeutic interventions, or invasive procedures are administered. As such, the anticipated risk to participants is minimal.

#### Adverse event monitoring plan

While adverse events (AEs) are not expected, the study team has established a system for the collection, assessment, and reporting of any solicited or spontaneously reported events that may arise in connection with study procedures, including but not limited to:

- Allergic reactions or discomfort associated with cycloplegic eye drops, if used
- Psychological discomfort or anxiety in younger children during assessments
- Unforeseen events related to data collection logistics (e.g., injury during on-site visit)

#### Collection and documentation

- Any untoward occurrence observed or reported by participants, parents, or school authorities during or immediately following data collection will be recorded in the Adverse Event Log, maintained by the site coordinator at AIIMS Bhopal.
- A trained ophthalmologist will evaluate the event and document the nature, severity, onset, outcome, and relatedness to study procedures.

#### Reporting and escalation

- All serious adverse events (SAEs), although unlikely, will be reported to the Institutional Ethics Committee (IEC) at AIIMS Bhopal within 24 h, followed by a detailed report within 7 days.
- The Principal Investigator will also notify the Trial Steering Committee during the next scheduled review, or immediately if the event is safety–critical.

#### Management and compensation

- Participants requiring follow-up care due to any AE deemed study-related will be referred to the Department of Ophthalmology at AIIMS Bhopal.
- Compensation, if applicable, will be provided in accordance with national ethical guidelines (ICMR 2017) and institutional policy.

### Frequency and plans for auditing trial conduct {23}

Although this is a minimal-risk, observational cohort study, periodic internal audits will be conducted to ensure protocol compliance, data integrity, and adherence to ethical and regulatory standards.

#### Outcome

Audits will be conducted quarterly throughout the 36-month study period and may be repeated more frequently if significant protocol deviations, safety concerns, or data inconsistencies are identified. Follow-up (re-audit) visits will be performed to verify implementation of corrective and preventive actions (CAPA), where applicable.

#### Audit frequency and scope

- Quarterly internal audit**s** will be conducted throughout the study period.
- These audits will include a review of:
  o Informed consent documentation
  o Participant enrolment logs and eligibility compliance
  o Data collection completeness and accuracy
  o Adverse event logs (if any)
  o Data storage and confidentiality procedures

#### Audit procedures

- Audit checklists will be developed in accordance with the approved protocol and Good Clinical Practice (GCP) guidelines.
- Each audit will include a random review of 10–15% of participant records.
- Discrepancies or protocol deviations, if identified, will be documented, addressed, and reported to the Principal Investigator and Trial Steering Committee.

#### Independence:

- Audits will be conducted by a designated quality assurance team from AIIMS Bhopal’s Research Oversight Unit, who are not directly involved in the day-to-day conduct of the study.
- These auditors are independent from the study investigators and the funding body, ensuring objectivity in monitoring compliance.

All audit findings and corrective actions will be documented in the Trial Master File (TMF) and will be made available to the Institutional Ethics Committee upon request.

### Plans for communicating important protocol amendments to relevant parties (e.g. trial participants, ethical committees) {25}

Any significant changes to the study protocol—including modifications to eligibility criteria, outcome measures, data collection methods, statistical analysis plans, or study timelines—will be formally documented, justified, and communicated to all relevant stakeholders in a timely and transparent manner.

#### Scope of amendments covered

- Changes in study objectives or endpoints
- Adjustments in participant eligibility criteria
- Modifications to data collection tools, timing, or procedures
- Updates to statistical methods or analysis populations
- Changes in site locations, study duration, or sample size

#### Notification and approval process

1. Principal Investigator (PI):
  Responsible for initiating and documenting any proposed amendment with justification.
2. Institutional Ethics Committee (IEC):
  All protocol amendments will be submitted to the IEC at AIIMS Bhopal for review and approval prior to implementation, in accordance with national ethical regulations.
3. Trial Steering Committee (TSC):
  Amendments will be presented to the TSC for advisory input and oversight.
4. Trial Registry:
  The trial registration entry on the Clinical Trials Registry of India (CTRI) or equivalent registry will be updated to reflect approved amendments, including changes in outcomes, methodology, or sponsor-related details.
5. Participating Institutions and Investigators:
  All study staff, including data collectors and site coordinators, will receive formal written communication outlining the approved changes along with training (if needed) to ensure implementation fidelity.
6. Participants (if applicable):
  In cases where the amendment directly affects participants’ rights, safety, or participation (e.g., changes in follow-up procedures or data usage), updated informed consent will be sought.
7. Publications and Sponsors:
  Journals, regulatory authorities (if applicable), and the funding agency (ICMR) will be notified of significant amendments at the time of submission or reporting.

### Dissemination plans {31a}

The investigators are committed to transparent, timely, and responsible communication of trial outcomes to all relevant stakeholders, including participants, the scientific community, healthcare professionals, policymakers, and the general public.

#### Communication to participants and schools

- Summary results will be shared with participating schools and parents/guardians via printed infographics or community reports written in clear, age-appropriate language.
- School-based dissemination events may be organized to explain the findings and their implications for student eye health and preventive strategies.

#### Scientific and professional dissemination

- The trial results will be submitted for publication in peer-reviewed open-access journals and presented at national and international ophthalmology, optometry, and public health conferences.
- Authorship will follow the ICMJE guidelines, and there are no publication restrictions imposed by the sponsor (ICMR).

#### Public access and registry reporting

- Summary results will be uploaded to the Clinical Trials Registry–India (CTRI) upon study completion, as per regulatory requirements.
- De-identified datasets, statistical code, and key findings may be made publicly available in open-access repositories (e.g., Zenodo, GitHub) in compliance with institutional policies and ethical approvals.

#### Policy and healthcare outreach

- Findings relevant to public health policy (e.g., screen-time guidelines, school eye screening protocols) will be shared with health authorities, NGOs, and educational boards to facilitate evidence-based interventions.

All dissemination efforts will respect participant confidentiality, ethical obligations, and intellectual property rights, and will aim to promote equitable access to study insights.

## Discussion

This study aims to address a significant and growing public health concern in India—the progression of myopia among school-going children—by developing an artificial intelligence–enabled predictive model based on longitudinal ocular and behavioral data. While the study is observational in nature and non-interventional, its scale, setting, and technological scope present several practical and operational challenges that merit consideration.

One of the primary logistical challenges lies in the school-based implementation of the study, which requires extensive coordination with educational institutions across both urban and rural areas in the Bhopal division. Securing administrative permissions, aligning data collection with academic calendars, and minimizing classroom disruption while maintaining compliance with ethical procedures (such as obtaining parental consent) are key operational priorities. The research team has incorporated several mitigation strategies, including liaison officers, institutional engagement workshops, and flexible scheduling of school visits.

The large-scale data collection effort, involving clinical ophthalmic measurements and behavioral questionnaires from 10,000 to 12,000 children, necessitates strong data management infrastructure. The use of real-time, tablet-based electronic data capture platforms with built-in validation rules addresses many quality assurance issues; however, variable internet access and field conditions may pose occasional risks to seamless data syncing and backup. Furthermore, standardization of measurements—especially cycloplegic refraction and axial length—is essential to model accuracy and will be supported by strict training protocols, inter-observer reliability checks, and central quality audits.

A significant challenge anticipated in the longitudinal phase is participant retention over the 24-month follow-up period. Migration, school dropouts, or parental withdrawal of consent may contribute to loss to follow-up. The study incorporates multiple strategies to mitigate this, including SMS reminders, parent-teacher engagement, and tracking systems to maximize follow-up compliance. Nevertheless, missing data remains a possibility and will be addressed through statistical techniques such as multiple imputation and sensitivity analyses [9, 10].

Conducting research in school-based settings may present practical challenges, including coordination with academic schedules, variability in parental consent return rates, and potential loss to follow-up due to student transfers or absenteeism. These challenges will be addressed through advance institutional coordination, flexible scheduling, structured parent engagement strategies, and systematic follow-up reminders to ensure minimal disruption and sustained participation [11, 12].

From a technological standpoint, developing and validating machine learning models that are both accurate and interpretable in a public health context is inherently complex. The study team has committed to using explainable AI approaches to ensure clinical utility and user trust. Another concern involves the ethical handling of large-scale, identifiable data from minors. The study has been designed in compliance with institutional and national data protection standards, with strict access controls and de-identification procedures in place. 

In conclusion, while the proposed study presents certain logistical and methodological complexities, its design incorporates structured mitigation strategies and oversight mechanisms. The integration of AI with epidemiological field research holds promise for scalable, evidence-based solutions to pediatric eye health challenges in low-resource settings.

## Trial status

- Protocol Version: Version 1.0
- Date of Finalization: 16 July 2025
- Date Recruitment Began: 01 August 2025
- Expected Completion of Recruitment: 31 March 2026

The recruitment timeline reflects the logistical considerations of conducting school-based data collection across multiple urban and rural sites in the Bhopal division. Follow-up assessments will continue for up to 24 months post-enrolment of the last participant.

## Data Availability

The final trial dataset will be accessible to the Principal Investigator (PS) and the designated members of the data management and analysis teams at AIIMS Bhopal and MANIT Bhopal. This includes authorized investigators involved in data analysis, model development, and manuscript preparation. There are no contractual agreements in place that limit or restrict access to the trial data for any of the investigators. The data will be stored securely on institutional servers, with access governed by role-based permissions and oversight from the Principal Investigator. De-identified datasets and statistical code may be made available for academic or policy research purposes upon reasonable request, subject to approval by the Institutional Ethics Committee and adherence to national data protection standards.

## Abbreviations

AI: Artificial intelligence
AIIMS: All India Institute of Medical Sciences
AUROC: Area Under the Receiver Operating Characteristic Curve
CBSE: Central Board of Secondary Education
CNN: Convolutional neural network
DMC: Data Monitoring Committee
eCRF: Electronic Case Report Form
FAS: Full analysis set
IEC: Institutional Ethics Committee
ICMJE: International Committee of Medical Journal Editors
MANIT: Maulana Azad National Institute of Technology
MCAR: Missing completely at random
MAR: Missing at random
MNAR: Missing not at random
SAP: Statistical analysis plan
SD: Standard Deviation
SER: Spherical equivalent refraction
SOP: Standard operating procedure
SPIRIT: Standard Protocol Items: Recommendations for Interventional Trials
TSC: Trial Steering Committee
